# Collaborative application of the food sustainability assessment framework (FOODSAF) to transform food systems and farmer livelihoods in Makueni County, Kenya

**DOI:** 10.1371/journal.pone.0342435

**Published:** 2026-04-21

**Authors:** Stellah Mikalitsa Mukhovi, Chinwe Ifejika Speranza, Boniface Kiteme, Aymara Zonta LIanque

**Affiliations:** 1 Department of Geography, Population, and Environmental Studies, University of Nairobi, Nairobi, Kenya; 2 Institute of Geography, University of Bern, Switzerland; 3 Centre for Training and integrated research in ASAL Development, Nanyuki, Kenya; 4 Leuphana University, Luneburg, Germany; Vivekananda Global University, INDIA

## Abstract

Food systems in arid and semi-arid lands face challenges that hinder them from meeting the food needs of communities and sustaining environmental resources. Using a collaborative approach between multiple societal stakeholders and academic actors, we applied the Food Sustainability Assessment Framework (FOODSAF) and a gender-differentiated participatory process in three workshops in Kenya involving women and men farmers, and social actors from the government, civil society, and private sector. The objectives of the study were to; a) assess the food sustainability of the Makueni agro-pastoral food system, b) assess the extent to which policies and practices of government, private sector, and civil society actors provide for an enabling or dis-enabling environment for farmers to achieve food sustainability, c) evaluate stakeholders perspectives of food sustainability indicators and identify areas in the food system that require intervention. The data were collected using workshops with farmers, policy implementers, private sector, and civil society actors, and analysed using descriptive analysis. The results show disparity in assessment of indicators by different stakeholders reflecting their perspectives, interests, experiences, and knowledge about each food system indicator. The performance of the value chain, the capacity of the food system to process and store food, and social self-organisation received the lowest scores and hence formed the basis for further reflection and collective action. This study shows that collective action supported by empirical evidence and collaborative considerations with stakeholders can contribute to the transformation of food systems and the associated farmer livelihoods. More collective actions are required to solve structural problems that smallholder farmers are incapable of negotiating individually.

## 1. Introduction

Food systems worldwide face pressures from global environmental change, declining soil fertility, population increase, institutional changes, erratic prices, land degradation, water stress, agrobiodiversity loss, among others [1,2,3]. Agropastoral food systems, especially in sub-Saharan Africa, are among the most vulnerable to climate change impacts including extreme events such as floods and droughts [4,5]. Since the World Food Summit in 1996, many countries are still struggling to eliminate hunger, which has been compounded by the COVID-19 pandemic, escalating food prices, fuel prices, and poverty [6] – an indication of unsustainable food systems. Major efforts in the United Nations Sustainable Development Goals (SDGs) have prioritized hunger and poverty reduction as goals to be achieved by 2030. However, achieving food and nutrition security, and securing incomes and reducing poverty, remain challenging [7,8]. Food systems may also have negative outcomes such as pollution by agrochemicals and the loss of biodiversity [9]. Sustainable production and consumption, structural policy interventions, and safety net programmes have been proposed as ways to achieve food security for the world’s vulnerable people [10,11].

Assessing food systems thus requires examining the input sources, how the food is produced, distributed, processed, retailed, consumed, and finally how wastes are managed [12,13]. In this study, food sustainability is conceptualized as consisting of five dimensions namely the right to food, social-ecological resilience, environmental performance, food security, and reduction of poverty and inequality [14]. A food system approach is a holistic framework that addresses the complex interactions and feedback between stakeholders, activities, and outcomes, and helps to identify intervention points to enhance peoples’ wellbeing [15]. The approach helps in balancing the economic, cultural, technological, political, social, and environmental aspects of food systems with a view of creating a sustainable future [16].

According to [17], the food systems approach enables the comparison of different intervention possibilities and to systematically analyse the synergies and trade-offs between different policy objectives. Considering that food systems vary from place to place and by individual experiences, it is important to adopt participatory instruments to assess food systems. One such instrument is the food sustainability framework that actors can use to identify and prioritize food system interventions. Several studies [18,19] have applied the food sustainability framework as a diagnostic tool to identify interventions with farmers through a participatory process. The FOODSAF was applied due to its strength as a learning and evaluation tool. FOODSAF enables farmers, practitioners and researchers to collaboratively learn about various aspects of a focus food system as well as identify bottlenecks to be addressed to make the food system more sustainable. The FOODSAF is a participatory tool allowing problem solving through multistakeholder discussions and consensus building [20]. In using a food system sustainability framework together with the community in a participatory process, we identify strengths and weaknesses of the focus food system and co-design interventions that can help create a more sustainable food system [21].

Using a systems theory, we apply the framework to the agro-pastoral food system in Kenya and assess the views of multi-stakeholders including policy implementers, private sector actors, smallholders, and civil society actors (S4 Table). We show how consensus was built by multiple stakeholders to arrive at an intervention to enhance food sustainability at the community level. Unlike other studies that looked at a single community, this study presents results of a collective action by three villages who agreed to work together to establish a grain aggregation store in one of the villages for mutual benefit. The intervention also included the establishment of an association to enhance the bargaining power of the members, collaboration, and collective decision making.

The objective of the study were to assess a) the food sustainability of the agro-pastoral food system of Makueni County, Kenya; b) the extent to which policies and practices of government, private sector, and civil society actors provide for an enabling or dis-enabling environment for farmers to achieve food sustainability, and c) to evaluate stakeholders’ perspectives of food sustainability indicators and identify areas in the food system that require intervention.

## 2. Materials and methods

### 2.1 The agro-pastoral food system in Makueni County, Kenya

Makueni County is located in the lowlands of Eastern Kenya at 1°48′S 37°37′E with a population of 949,298, comprising 49% males and 51% females (Kenya National Bureau of Statistics [Kenya National Bureau of [22]. It has an area of about 8,008.9 km² with rainfall between 300–400 mm (mainly in the lowlands) received in two rainy seasons, while temperature of about 35^o^C is experienced in the low-lying areas of the county. The livelihoods of most county residents depend on small-scale farming that is dominantly rainfed with maize, green grams, pigeon peas, cowpeas, sorghum, pumpkin, sweet potatoes and fruits; mangoes, pawpaw, and oranges as main crops grown on average land holding of 2–4 acres [19]. The main markets for pigeon peas are USA and India, and locally in main towns, while green grams are sold mainly in East Africa and Southern African countries [23]. The farmers integrate crop production with cattle, goats, and poultry.

The county is characterized by high poverty levels with a multidimensional poverty rate of more than 50% compared to the national absolute poverty level (47%) [24]. Poverty in the county is most severe in the dry areas of Kathonzweni, Mavindini, Kithuki, Kalawa, and Kitise. The high poverty level is as a result of several factors, the main one being low agricultural productivity (mainly due to water scarcity, and poor soils), poor access to markets, high unemployment rates, and climate change vulnerability [25,26].

### 2.2 Methods

#### 2.2.1 The food system sustainability assessment framework.

FOODSAF is an assessment framework developed in the frame of a transdisciplinary multi-case study research project over a six-year period (2015–2021) to assess the extent to which food systems meet the five dimensions of food sustainability; the right to food, social-ecological resilience, reduction of poverty and inequality, food security, and environmental performance [27]. For each dimension of FOODSAF, we selected 3 indicators that gave us a total of 15 indicators (Table 1). The framework enabled us to use a participatory process with stakeholders to identify the strength and weaknesses of the food system and to identify possible interventions. The framework was developed by various academic and non-academic actors within ‘*the towards food sustainability project”* funded by the Swiss National Science Foundation and initially implemented in Kenya and Bolivia and later extended to Zambia, Ghana, Brazil and Colombia [28]. It promotes co-creation of knowledge together with stakeholders, connects science to transformative processes and has potential to influence policy that contributes to sustainable food systems.

**Table 1:** Summary of FOODSAF assessment by different stakeholders.

FOODSAF Dimensions and Indicators	Scoring by government officers	Scoring by private sector and civil society actors	Scoring by women farmers	Scoring by men farmers
Food security	3	3.6	3	2
Household food security	3	4	3	3
Power relations (regarding food in	3	3	3	2
household and in the food system)				
Capacity of the food system to store and process food	3	4	3	1
Right to food	4	3.3	4.3	2.6
Non-discrimination	4	3	4	2
Access to information	4	4	5	4
Effective participation	4	3	4	2
Poverty and inequality	2.7	3	2.6	1.7
Sources and levels of incomes	2	2.3	3	2
Access to socio- technological infrastructure	3	3	3	2
Performance of food value chains	3	4	2	1
Environmental performance	4	3.7	3.7	2.7
Environmental benefits of the food system landscape	4	4	3	2
Carbon footprintsequestration (Trees in the food system)	4	4	4	4
Impact on human health	4	3	4	2.3
Social-Ecological Resilience	3.3	3	3.7	2.3
Diversity	4	3	3	3
Social self- organisation	4	4	2	4
Ancestral knowledge (Indigenous and Local Knowledge)	2	2	4	2

#### 2.2.2 Applying the FOODSAF in participatory workshops.

**Fig 1:**
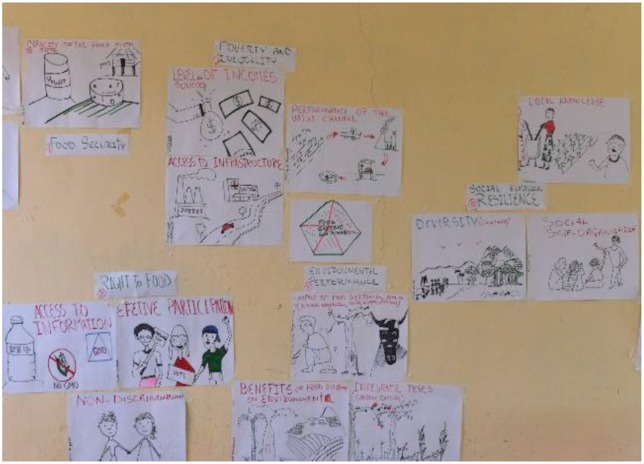
Drawings showing some FOODSAF indicators and scoring criteria.

**Fig 2:**
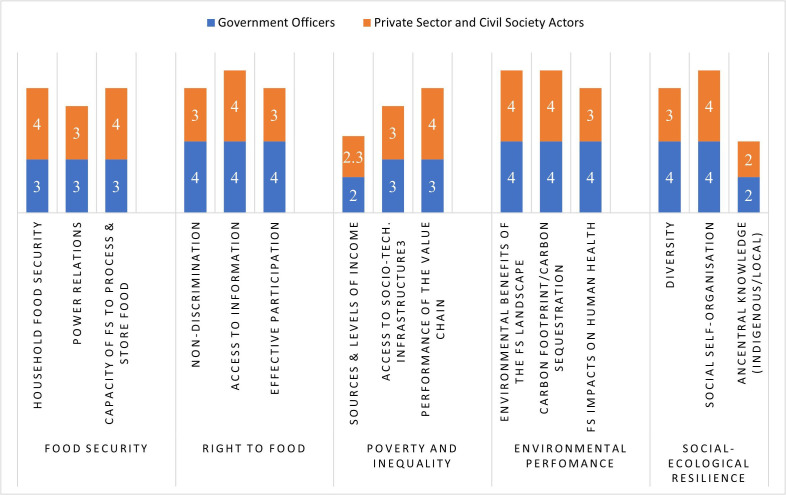
Assessment of indicators by government officers, and private sector and civil society.

FOODSAF was conducted in three participatory workshops in 2019 to identify potential interventions using a multi-stakeholder approach while the implementation phase was completed in 2022. To address the first objective, a participatory workshop with farmers was used to assess from their perspectives, the sustainability of their agro-pastoral food system using the FOODSAF indicators (S1 Fig). The 19 farmers comprising 10 women and 9 men, were drawn from three villages: Kwakavisi, Mbuvo, and Mavindini located in the three wards Kathozweni, Kitise/ Kithuki, and Mavindini respectively. Using purposive sampling, we selected the most active members from farmer groups through the help of county extension personnel. To capture gender perspectives, the farmers were divided into men and women farmers’ focus group discussion. The facilitators comprised researchers and practitioners engaged in the multi-case study research project and extension personnel from Makueni county who were involved in the entire process of the transformative pilot project to ensure the sustainability of the intervention. The different dimensions of the FOODSAF were explained and discussed with all farmers in one session using drawings (Fig 2) prior to the assessments using the local Kikamba language (two of the facilitators spoke the local Kikamba language). After ensuring the farmers understood the dimensions, a Likert scale of 1–5 (1-very bad to 5- excellent) (Fig 1) was used by the groups to score each indicator. Each group was accompanied by two researchers/facilitators that recorded the discussions leading to the scores assigned to each dimension and the associated explanations. After the assessments, we compared and discussed the scores of men and women farmers thereby highlighting areas of divergence and convergence and discussing options for transformative actions to improve the food sustainability of the agro-pastoral food system. The FOODSAF assessment guide was used throughout the process while the discussions were captured using a tape recorder as well as notes.

We conducted another similar participatory workshop with practitioners from Government (6), private sector (5), and civil society (5) using the same scoring criteria to address the second objective, also using the FOODSAF (S2 Fig). The practitioners were representatives of organisations that were actively engaged in agriculture and food security in the county. We adopted a multi-level and polycentric governance perspective that includes policies and activities (practices) at the county and national levels, as well as considering the influences of both government and non-government actors on the food system. We divided the participants into 2 groups, one for government actors (Extension officers in the county) and the other for both the private sector and civil society actors (e.g., Purchasers for industry, international NGOs such as World Vision). We asked the group of government officers comprising mainly, extension officers, to assess to what extent government policies and practices (activities) provide for an enabling or dis-enabling environment for farmers to achieve food sustainability. We posed the same question to the group comprising private sector and civil society practitioners to assess the contributions of their sector to food sustainability in the county. We then not only compared the scores given by the two groups of practitioners, but also those scored in the previous workshop with men and women farmers. In the third workshop, the discussions were aimed at building consensus by all actors on the best collective action to address the challenges identified by stakeholders (S3 Table). We again discussed the convergence and divergence in the scores of the four groups and identified potential transformative actions to improve the sustainability of the agro-pastoral food system and follow-up actions.

## 3. Results

### 3.1 Smallholders perspectives on food sustainability

The results show that both men and women agreed on access to information, household food security, carbon footprint/sequestration, source and levels of income, diversity, and household food security (Table 1). According to women farmers, social self-organisation was good (4) while men farmers rated it as bad (2) (S3 Table). The rest of the actors agreed with women's assessment on social self-organisation as being good (4). The high score by women farmers on social self-organisation could be linked to their role in Self- Help Groups (SHG) locally known as *chamas* which are often dominated by women. Women also scored some indicators high compared to men, such as the capacity of the food system to store and process food (men 1, women 3).

Regarding overall average assessment of the indicators by farmers, the highest score was on the right to food by women (4.3 very good) and the lowest was on poverty and inequality as scored by men (1.7 bad). There was agreement about the average assessment scores for men and women on environmental performance where the scores were 3 (women) and 3.3 (men). The farmers gave reasons such as high adoption of agroforestry, implementation of soil and water conservation measures, conservation agriculture, and use of organic fertilizers.

The indicators that were rated highly by extension personnel were diversity (4), self-organisation (4), integration of trees into agricultural landscapes (4), environmental benefits of the food system (4), and impacts of food systems on health (4) (Table 1 and Fig 2). The lowest scores were: use of indigenous and local knowledge (2), and sources and levels of income (2). Regarding mean scores, the highest was the right to food (4), meaning that access to information, effective participation, and non-discrimination were all rated highly. On the contrary, poverty and inequality received the lowest mean score of 2.7 an indication that the food system actors, activities, and processes did not help to reduce poverty and inequality.

**Fig 3:**
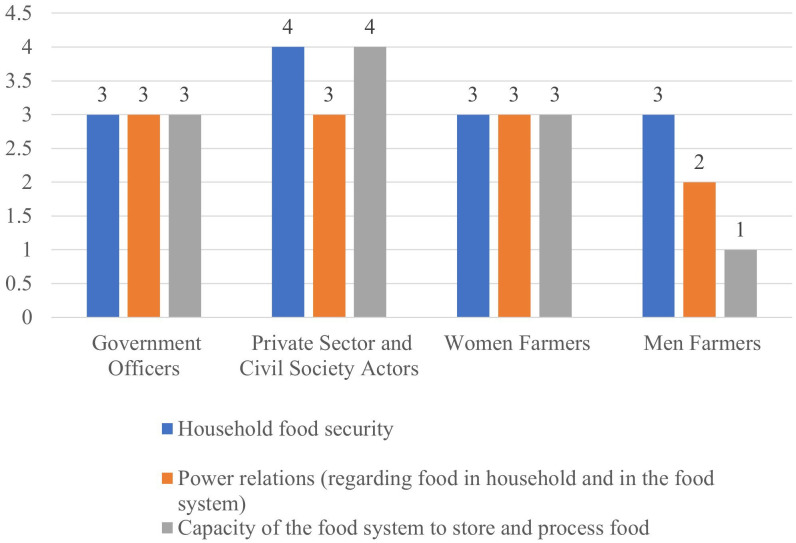
Stakeholder perspectives on food security.

**Fig 4:**
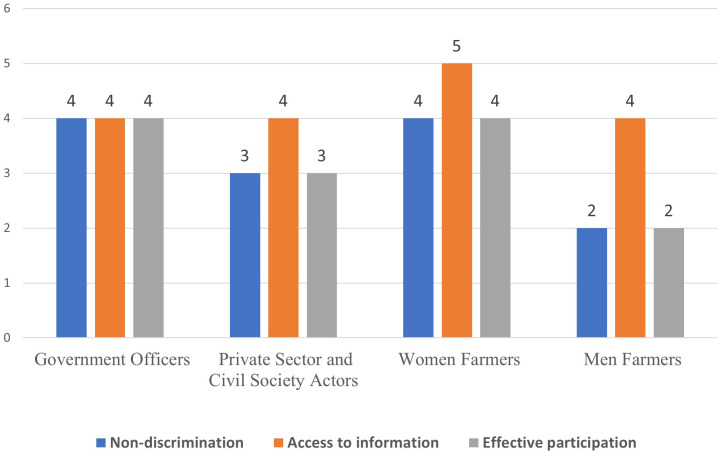
Stakeholder perspectives on the right to food.

The civil society actors agreed with the extension personnel on local knowledge but not on diversity, they also disagreed on food system impact on health where extension personnel gave a high score of 3 while civil society gave a low score of 2. Additionally, “sources and levels of income” was given a high score by extension officers but a low score by civil society (Fig 3). The highest mean score for civil society was 3 for all the five indicators (Table 1 and Fig 4).

### 3.2. Assessment of FOODSAF indicators and identification of areas for improvement

#### 3.2.1 Food security.

The highest possible score of 4 (good) was by the private sector and civil society on household food security and capacity of the food system to process and store food (Fig 3). The private sector actors argued that the households have a diversity of foods, and that they use traditional methods to process food. Women are more involved in food processing and storage than men [29,30], hence their view may reflect a better understanding regarding this indicator. The government officers and women farmers agreed on all indicators while men farmers gave the lowest score of (1) to the capacity of the food system to process and store food.

#### 3.2.2 Right to food.

Government officers and women farmers gave high scores for all indicators (effective participation, non-discrimination and access to information) while the men farmers gave a low score of 2 (bad) for non-discrimination, and effective participation (Fig 4).

Women gave the highest score of 5 (excellent) for access to information which contradicts previous studies that have shown that access to government extension services in the county is poor [31],however, there are possibility of alternative sources of information/extension service that farmers access. Article 43 (1) (c) of the Constitution of Kenya, 2010, on the Social and Economic Rights states that ‘*every person has a right to be free from hunger, and to have adequate food of acceptable quality*’ thus providing for a human right-based approach to food security in Kenya. This is in line with international standards emanating from the 1966 International Covenant on Economic Social and Cultural Rights (ICESCR) Article 11.2 that recognizes the fundamental right of everyone to be free from hunger and identifies corresponding state obligations [32,33].

Nine years after the promulgation of the Kenyan constitution, evidence from the grassroots show that efforts exist on the ground to achieve the right to food. This is related to high scores given by the government officers who perhaps have a better perspective of the concept and efforts to achieve it. Obligation to fulfil the right to food places a duty on governments to facilitate access to and to provide food to the vulnerable people in the society. The farmers also mentioned receiving relief food during severe droughts and that those who were 65 years and above and vulnerable, were enrolled on the government cash transfer program [34,35].

#### 3.2.3. Environmental performance.

**Fig 5:**
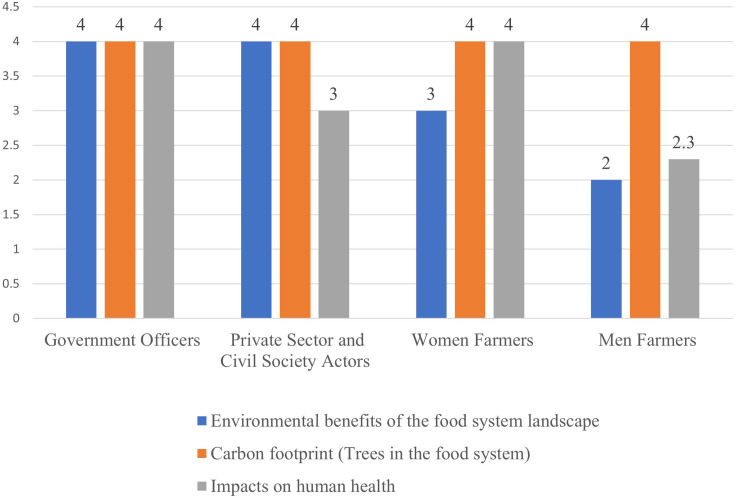
Stakeholder perspectives on environmental performance.

Environmental performance received high scores from almost all the actors except men who rated environmental benefits of food system landscapes and impacts of the food systems on health as bad (Fig 5).

The reasons given were limited knowledge of agrochemicals and their excessive use that is likely to affect the health of farmers. On the other hand, government officers, civil society actors, and women rated all the indicators as either fair or good. They gave various reasons: integration of crops with trees, control of soil erosion by terracing, integrating agrochemicals with organic farming among others. These arguments support studies in Makueni County that show success in the implementation of Soil and Water Conservation (SWC) measures practiced for several decades [36]. Most farmers practice contour ridging, agroforestry, and other in situ rainwater conservation by trapping water and preventing runoff. Farmers in Makueni and Machakos practice diverse SWC technologies on their farms. The most popular practices are cut-off drains, retention ditches, terracing, run-off harvesting, and agroforestry. Additionally, Makueni farmers have also embraced Conservation Agriculture (CA) [37].

#### 3.2.4 Poverty and inequality.

**Fig 6:**
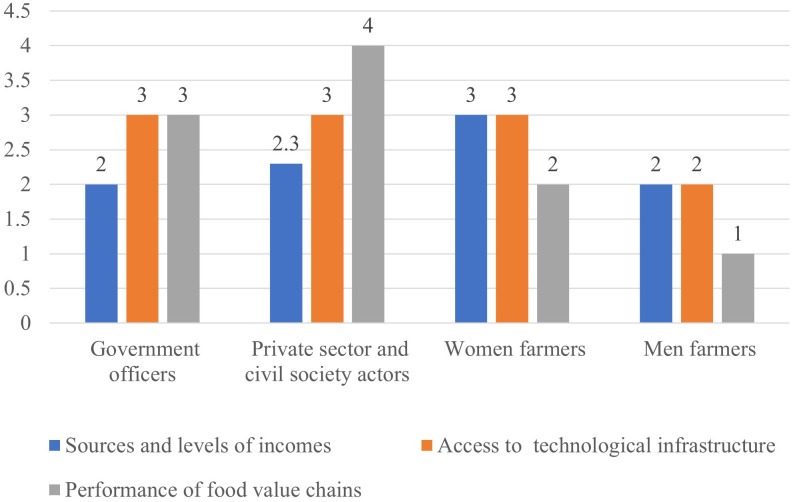
Perspectives on poverty and inequality in the food system by government officers, private and civil society actors.

"Sources and levels of income was scored low (2) by many actors except for women farmers who scored it as fair (3). On the other hand, civil society actors gave a high score of good (4) for performance of the value chain while other actors- women and men farmers and government actors gave a score of 3 and below (Fig 6). During the discussions, performance of the value chain was perhaps the most contentious issue since the farmers complained about intermediaries (also referred to locally as middlemen or brokers) exploiting them with low prices. This was attributable to poor post-harvest management that compeled farmers to dispose of their farm produce immediately at low prices. Indeed, studies have shown that smallholder farmers’ resilience is affected by poor producer prices due to many factors including exploitation by middlemen [38]. Poor infrastructure could also contribute to low prices, leading to overall low scores of poverty and inequality. The farmers also argued that roads are available but in poor condition during the rainy seasons, constraining their access to markets.

#### 3.2.4 Social-ecological resilience.

**Fig 7:**
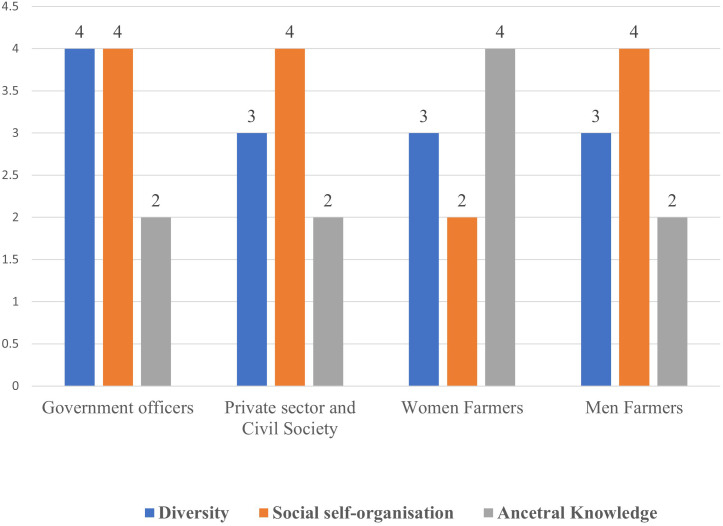
Stakeholders perspectives on social-ecological resilience.

The highest score of 4 (good) was diversity (government officers), social-self organisation (civil society and women), and use of ancestral knowledge (indigenous and local knowledge (women) (Fig 7). Apart from women farmers, all the other actors gave a score of 2 (bad) for use of ancestral knowledge. The reasons by women for high scores on indigenous knowledge were that they eat traditional foods, some still use traditional utensils-calabashes and gourds, use traditional production practices, and indigenous and local knowledge in post-harvest management combined with strategies such as agroforestry. Social self-organisation was rated bad (2) by women farmers, but good (4) by private and civil society actors, government officials, and men (S4 Table). On the other hand, the high score by government officers (4- good) could be an indication of the reality as SHGs are registered by the State Department for Gender and Affirmative Action

### 3.3 Synthesis of the views from multi stakeholders’ and prioritization of food system interventions

**Fig 8:**
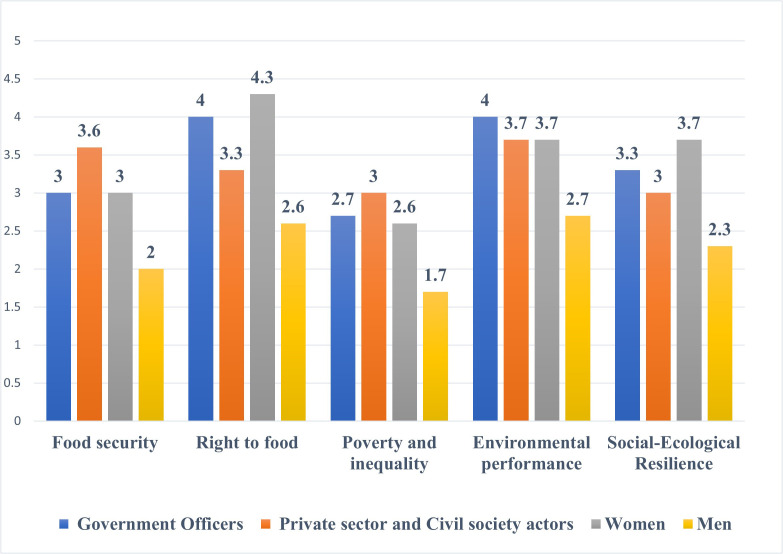
Summary of assessment by all stakeholders.

The purpose of conducting the participatory food sustainability assessment was to identify the weak areas in the food system and to prioritize them for intervention purposes. The dimensions that received the lowest mean scores were poverty and inequality, followed by food security (Fig 8). The highest scores were right to food, and environmental performance of the food system (Table 2). It therefore, meant that possible areas of interventions were to target the dimension of poverty and inequality, and food security (Table 2). Under food security, the most critical area that participants identified was the *low capacity of the food system to process and store food,* while under poverty and inequality, it was *poor performance of the value chains*.

**Table 2:** Prioritization of interventions by stakeholders (Higher mean food sustainability score means less need for intervention; Rank: 5 means need for intervention, 1 means less need for intervention).

Dimension	Mean food sustainability score	Rank	Specific area of Intervention areas	Interventions
Right to food	3.5	1	No intervention	No intervention
Environmental performance	3.3	2	No intervention	No intervention
Social-ecological resilience	2.9	3	Social-self- organisation	Due to low social self-organisation, the farmers agreed to form a Community Based Organisation CBO called MAKWAMBU whose officials would oversee the grain store
Food Security	2.8	4	Capacity of food system to process and store food	Creation of a grain aggregation store for bulking and selling directly to the market addressed the two challenges
Poverty and inequality	2.5	5	Performance of the value chain	Creation of a grain aggregation store enabled farmers to sell in bulk directly to the market

The consensus building workshop embarked on negotiations, consultations, and benchmarking on interventions together with stakeholders in the county. This was geared towards identifying best interventions that could reduce postharvest losses, ensure smooth marketing of farm grains, and improve income for farmers. After lengthy negotiations, the stakeholders agreed to come up with two interventions to simultaneously address the two challenges: poor performance of the value chain, and the low capacity of the food system to process and store food. The challenge of poor performance of the value chain and low capacity of the food system to process and store food was addressed by one solution – the construction of a grain aggregation store to support direct marketing of grains instead of passing through intermediaries (Table 2).

The grain aggregation store served several purposes: a) reduced post-harvest losses-associated with pests and aflatoxin, b) enabled farmers to sell in bulk, c) improved farmers’ bargaining power, d) enhanced members’ livelihoods by ensuring better income for farm produce, e) transformed post-harvest management for grain in the three villages, f) improved prices for grains, g) improved seed security because the farmers stored their seeds, and i) improved income due to reduction of losses, selling direct to the market, reduced sale of grain for short term needs, and associated benefits for livelihood security. Additionally, grain bulking and storage for commercial purposes had potential to attract young and older men who have traditionally perceived the role of post-harvest management as a woman’s activity. In order to sustain the collective action, the officials of the CBO created by the collective action were charged with the responsibility of operating the store, but also other activities such as funds mobilization for the CBO, savings and loans association for members, table banking, environmental stewardship activities, and income generating activities for members. The CBO worked closely with an extension officer from the county who was involved in the collective action right from the initiation stage.

Even though the scores for social-self organisation were high (4) for all actors except men (2), the mean score for social-ecological resilience was 2.9 compared to lowest score of 2.5 for poverty and inequality. Participants agreed through consensus to form a community organisation to oversee the running of the store after project closure. In this regard, a Community Based Organization (CBO) bearing the names of the three villages **Ma**vindini, **Kwa** kavisi and **Mbu**vo was formed (MAKWAMBU) and registered with the State Department for Gender and Affirmative Action. The role of the CBO was to enable the farmers from the three villages to collectively address the challenges facing them, oversee the running of the grain store, marketing of the grain, and to spearhead the vision of the CBO.

## 4. Discussion

This analysis illustrated the systemic character of food. From production to consumption, food involves various actors with different interests and strategies that determine whether a food system achieves its goals. Such goals include being environmentally sustainable, social-ecologically resilient, supporting livelihoods, food and nutrition outcomes, and the reduction of poverty and inequality [39]. Thus, processes in one dimension of a food system can have system-wide effects on the whole food system but depends also on the interactions with other food system components. In this study, the policy of the right to food in Kenya did not automatically translate to positive food system outcomes, although it might serve as a criteria or benchmark to assess food system interventions [40]. The elaborations by workshop participants also show the role of history in current food systems as colonial legacies may have positive or negative effects on current food systems [41,42].

Comparing the perspectives of men and women reveals how their roles in the household influence their perspectives about the food system. For instance, women play a critical role in food production, processing, marketing, and household provisioning [43] hence their perceptions on some of the indicators such as access to information, diversity and food security reflected their experience in these roles. Women’s high scores on access to information could result from; a) their role as farm managers because their husbands mostly migrate out of the village to look for job opportunities, b) their membership in Common Interest Groups that are targeted by extension officers who use a group approach in offering services, c) and besides the formal extension services, women use informal information sources such as other farmers, NGOs, and the private sector [44,45]. On the other hand, men’s lowest score on capacity of the food system to process and store food and performance of the value chain could be related to their decision-making roles at the household level [46,47]. As much as women were involved in the actual production, men made the decisions on the types of crops to be planted, the acreage, and after harvest, how much produce was to be stored and sold. Men were hence familiar with prices of agricultural commodities and market prices. Income from the farms was critical in meeting household needs such as healthcare, and children’s education [48].

The high scores for the environmental performance were as a result of decades of campaigns by both government and NGOs to restore degraded lands in the larger eastern region of Kenya through soil and water conservation measures. Such measures included agroforestry, terraces, grass strips, soil bunds, and conservation agriculture among others [49]. Makueni is vulnerable to drought-induced food insecurity while other risks such as climate change and variability have been identified to affect smallholder farmers livelihoods and land resources [50]. Efforts to address severe land degradation in several counties in the lower eastern part of Kenya begun in 1990s [51]. NGOs operating in the study region often focus on promoting sustainable land management. Success in soil conservation as observed by the farmers and other stakeholders is attributable to historical factors -programmes on soil conservation and soil erosion control introduced by the colonialists and later institutionalised by the second president of Kenya [52].

Poor performance of the value chain was due to various factors: low value addition, control of markets by brokers (middlemen), low levels of commercialization, low productivity, fragmented markets, low investment in postharvest technologies, poor infrastructure, and vulnerability to climate change [53], [Muia et al., 2024]. Addressing the above challenges can improve the value chain performance leading to multiple benefits for the households. Poor performance of the value chain, among other factors, contributes to low income and food insecurity of the producers. Other factors include a) an insecure land tenure system, poverty, low levels of commercialization, inadequate policies, and limited investment in irrigation [54,55,56].

The right to food received highest scores by all stakeholders, showing involvement of the state and non-state actors’ in guaranteeing the right to food for the most vulnerable people in society. Several vulnerable families received cash transfers from government. These included old people above 65 and vulnerable, people living with disability, and orphan headed households. Persons enrolled in the cash transfer program received Ksh. 2000 per month (15.3USD) [57]. Government interventions such as cash transfers and other safety net programmes helped to meet the household needs of vulnerable persons in society and build resilience against shocks. However, the sustainability and governance of such interventions have been questioned [58,59,60]. In addition, due to the high vulnerability to severe droughts in the region, families also received relief food during droughts, among other household and community adaptation measures [61,62]. It is important to note that this research was affected by COVID-19 pandemic, and the vulnerable households in Kenya and other Countries in Sub-Saharan African were supported by cash transfer that was made possible through donor funding [63,64]. Local collective action also has potential to transform the food systems with better outcomes for food security and livelihood resilience [65].

While FOODSAF has been co-created through transdisciplinary research involving both academic and non-academic actors, it has some limitations: It a) assumes that the academic and non-academic actors involved have profound previous knowledge of the food system being assessed; b) requires that the indicators are domesticated to the context being assessed; c) requires more time for the actors to comprehend the framework before the assessment of the food system, and d) in cases, where no prior research and deep knowledge of the food system exists, it may require a joint fieldwork of academic and non-academic actors to first understand the food system before assessing it. Despite these limitations, FOODSAF has been applied in different contexts and food systems to diagnose challenges and prioritize interventions in six countries (three in Africa, and three in South America) [66].

The outcome of 15 projects arrived at through participatory processes by stakeholders is an indication of its strength. The strength of the framework is also in its participatory nature allowing multi-stakeholders from grassroots to policy level to collaboratively determine development pathways for a community. In addition, the framework focuses on consensus building which is critical for the ownership and sustainability of collective action. A participatory process has several strengths: it a) enables decisions to be taken promptly, b) incorporates diverse perspectives, c) enhances ownership of the collective action, d) enables learning among stakeholders, and e) it helps to build trust among community members and between a community and researchers [67]. However, a participatory process is time consuming, resource intensive, and may be dominated by certain groups [68,69].

## 5. Conclusion

This study applied the FOODSAF tool to the agro-pastoral food system of Makueni county to assess the performance of the food system in five dimensions (right to food, social-ecological resilience, reduction of poverty and inequality, food security, and environmental performance) and to identify levers to enhance the sustainability of the food system. The study confirms that stakeholder perspectives are important in understanding the challenges facing food systems and designing lasting solutions through collective actions. Using the FOODSAF in two gender-differentiated and two sectorial-stakeholder workshops and bringing all stakeholders (participants) together in a consensus workshop to identify and initiate intervention priorities to improve the food system, shows the importance of FOODSAF as an effective diagnostic instrument. Three major concerns that needed urgent solutions were identified, poor performance of the value chain, low capacity of the food system to process and store food, and low levels of social self-organisation. While there are many ways of addressing value chain bottlenecks, all the stakeholders supported the establishment of a grain aggregation store to enhance the farmers’ food system. The FOODSAF can thus be applied to other food systems with the relevant contextual adjustments. Further research steps could entail evaluating the effectiveness of the initiatives identified through the FOODSAF to further advance learning about the sustainability of food systems.

## Supporting information

S1 Fig Indicators used in food system assessment. (PDF)

S2 Fig Participatory scoring of indicators by different actors. (PDF)

S3 Table Justification of the scores by women farmers. (PDF)

S4 Table Actors in Makueni agropastoral food system. (PDF)
