# Supplementary figures and images for: Collaborative application of the food sustainability assessment framework (FOODSAF) to transform food systems and farmer livelihoods in Makueni County, Kenya
Source: PLoS One. 2026 Apr 21;21(4):e0342435. doi: 10.1371/journal.pone.0342435 (PMC13099092; doi:10.1371/journal.pone.0342435)

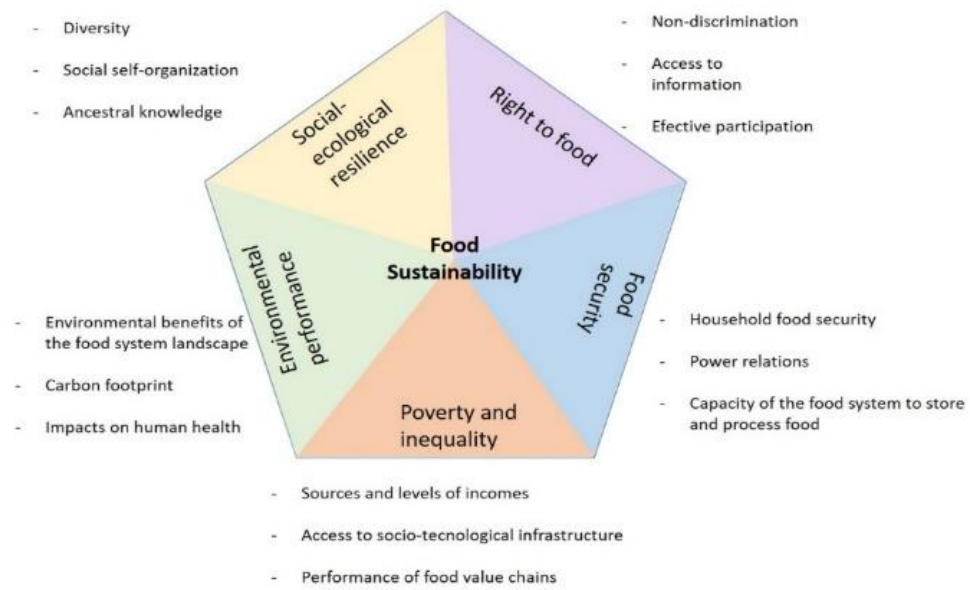

### S1 Indicators used in food system assessment in makueni

Supplement: S1 Fig — (PDF) [file pone.0342435.s001.pdf]

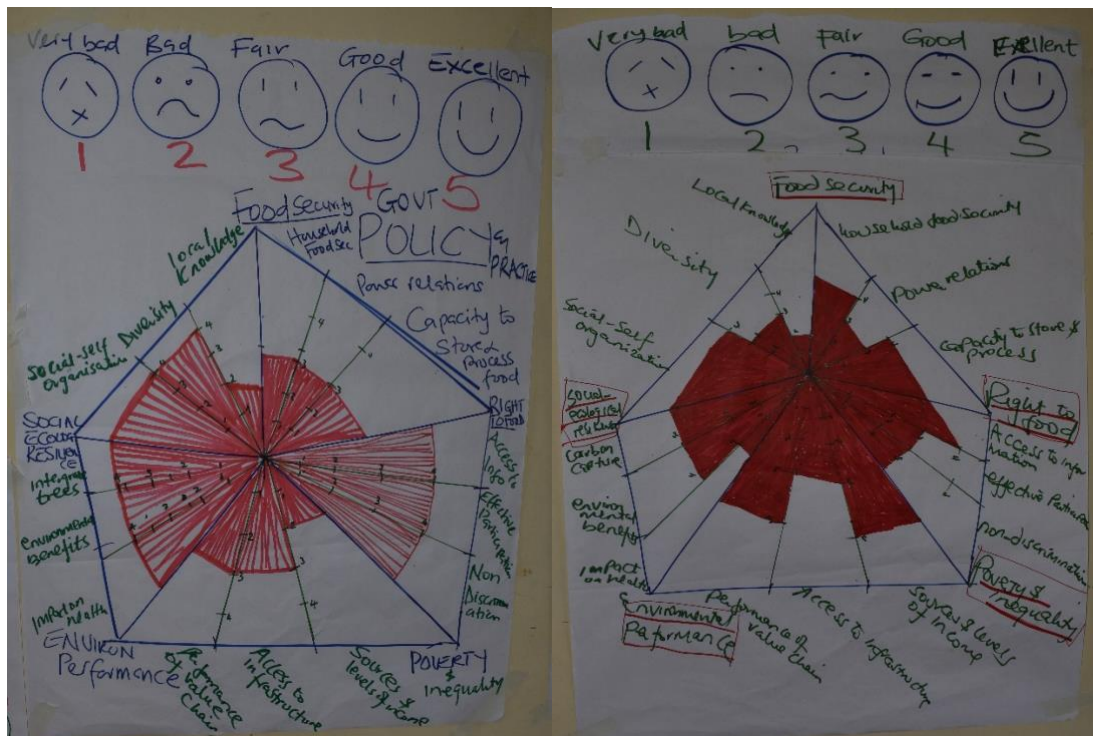

S2: Participatory assessment of the food system by different actors

Supplement: S2 Fig — (PDF) [file pone.0342435.s002.pdf]
