# Supplementary material for: Collaborative application of the food sustainability assessment framework (FOODSAF) to transform food systems and farmer livelihoods in Makueni County, Kenya
Source: PLoS One. 2026 Apr 21;21(4):e0342435. doi: 10.1371/journal.pone.0342435 (PMC13099092; doi:10.1371/journal.pone.0342435)
Supplement: S3 Table — (PDF) [file pone.0342435.s003.pdf]

S3: Reasons/justification for the scores by women

	Indicators per Dimension	SCORE	Reason for the score
	FOOD SECURITY		
1	Household food security	3	Not all households can afford good and enough food all the time.
2	Power relations	3	People are not really equal but relatively no big differences.
3	Capacity of food system to store	3	Sometime they dry green cowpeas leaves for vegetables to use later, they mill maize to flour, and they store maize and legumes for future use.
	RIGHT TO FOOD		
4	Access to information	3	There is enough information on the food they eat – mostly own production, from neighbors and local markets, including seeds
5	Effective participation	4	They participate well in most forums discussing food issues in the area, they are involved in community activities
6	No discrimination	4	There is no discrimination in the community
POVERTY AND INEQUALITY			
7	Levels of income sources	3	Few do weaving for income, majority sell farm produce and livestock products for income
8	Access to infrastructure	3	Many infrastructures available, though some not in good condition e.g. roads, schools. Roads are mussy during the rainy season, Many areas connected to power
9	Performance of value chain	2	Production is well done but storage is a problem, markets not available or are poor.
	ENVIRONMENTAL PERFORMANCE		
10	Benefit of food system on environment	4	They do SWC, water harvesting, plant trees for oxygen and clean air, practices good friendly farming practices e.g. using manure. They are interested in recovering the landscape, producing food.
11	Impact of food system on health	3	Use of herbicides in CA affects soils and vegetation and sometimes get into food/fruits/livestock causing health issues. Sometimes Maize crop is affected by aflatoxins.
12	Integration of trees (carbon capture)	4	Many have planted trees both exotic and indigenous on their farms as sources of income
	SOCIAL- ECOLOGICAL RESILIENCE		
13	Self-organization	2	Less community organization in the area.
14	Diversity (environment, crops, livestock)	3	There are trees, different fruits, livestock, and water – all in fair portions. As a result of CA (for example: intercropping), the landscape is more resilient.
