# Supplementary material for: Collaborative application of the food sustainability assessment framework (FOODSAF) to transform food systems and farmer livelihoods in Makueni County, Kenya
Source: PLoS One. 2026 Apr 21;21(4):e0342435. doi: 10.1371/journal.pone.0342435 (PMC13099092; doi:10.1371/journal.pone.0342435)
Supplement: S4 Table — (PDF) [file pone.0342435.s004.pdf]

S4 :Actors in Makueni food system

Actors	Actors Roles
**Individual farmers**	Production, processing, transportation and marketing of farm produce
**National Cereals and Produce Board**	commercial trading in grains,<br>grain handling and storage<br>maintaining Strategic Food Reserve<br>Famine Relief in collaboration with national and county governments,<br>procuring and marketing high quality farm inputs
**Brokers/Middlemen**	Collecting and transportation of farm produce to various markets
**Kenya Bureau of Standards (KBS)**	Quality assurance and inspection<br>Market surveillance<br>Testing services
**Ministry of Agriculture, Livestock and Fisheries**	Formulation of policies in dairy value chain
**Agriculture Sector Development Support programme (ASDSP)**	Value chain development<br>Capacity building
**Kenya Agricultural Productivity Programme (KAPP)**	Value chain development
**County government**	Capacity building through ATCs<br>Value addition
**Kenya Agriculture and Livestock Research Organization (KALRO)**	Research in drought tolerant crops<br>Capacity building<br>Link with extension personnel for research dissemination
